# MicroRNA-146a: A Comprehensive Indicator of Inflammation and Oxidative Stress Status Induced in the Brain of Chronic T2DM Rats

**DOI:** 10.3389/fphar.2018.00478

**Published:** 2018-05-14

**Authors:** Yangmei Xie, Aiqun Chu, Yonghao Feng, Long Chen, Yiye Shao, Qiong Luo, Xiaolin Deng, Men Wu, Xiaohong Shi, Yinghui Chen

**Affiliations:** ^1^Department of Neurology, Jinshan Hospital, Fudan University, Shanghai, China; ^2^Department of General Medicine, Shihua Community Health Service Center, Shanghai, China; ^3^Department of Endocrinology, Jinshan Hospital, Fudan University, Shanghai, China; ^4^Department of Neurology, Huashan Hospital North, Fudan University, Shanghai, China

**Keywords:** microRNA-146a, type 2 diabetes mellitus, brain impairment, thymoquinone, inflammation, oxidative stress, biomarker

## Abstract

**Objective:** It was demonstrated that inflammation and oxidative stress induced by hyperglycemia were closely associated with alteration of miR-146a. Here, we investigated the role of miR-146a in mediating inflammation and oxidative stress in the brain of chronic T2DM rats.

**Methods:** The chronic T2DM (cT2DM) models were induced by intraperitoneal administration of STZ (35 mg/kg) after being fed a high-fat, high-sugar diet for 6 weeks. H&E staining was conducted to observe the morphological impairment of the rat hippocampus. The expressions of inflammatory mediators (COX-2, TNF-α, IL-1β) and antioxidant proteins (Nrf2, HO-1) were measured by western blot. The levels of MDA and SOD were detected by the respective activity assay kit. The levels of p22phox and miR-146a were examined by quantitative real-time PCR (qRT-PCR). The expressions of IRAK1, TRAF6 and NF-κB p65 were measured by western blot and qRT-PCR. Pearson correlation analysis was performed to investigate the correlations between miR-146a and inflammatory mediators as well as oxidative stress indicators.

**Results:** The expression of miR-146a was negatively correlated with inflammation and oxidative stress status. In the brain tissues of cT2DM rats, it was observed that the expressions of inflammatory mediators (COX-2, TNF-α, IL-1β) and oxidative stress indicators including MDA and p22phox were elevated, which were negatively correlated with the expression of miR-146a. While, the antioxidant proteins (Nrf2, HO-1, SOD) levels decreased in the brain of cT2DM rats, which were positively correlated with the miR-146a level. The expressions of NF-κB p65 and its specific modulators (IRAK1&TRAF6) were elevated in the brain of cT2DM rats, which might be inhibited by miR-146a.

**Conclusion:** Our results implied that increased inflammation and oxidative stress status were associated with brain impairment in cT2DM rats, which were negatively correlated with miR-146a expression. Thus, miR-146a may serve as a negative comprehensive indicator of inflammation and oxidative stress status in the brain of chronic T2DM rats.

## Introduction

Type 2 diabetes mellitus (T2DM) is one of the most common endocrine disorders, which has already gained high public attention in the past few decades. The clinical complications of T2DM, like cardiovascular diseases, neuropathy, nephropathy, and retinopathy seriously affected the survival and life quality of patients with T2DM (Sims-Robinson et al., 2016). Recently, dysfunction of the brain induced by T2DM has emerged as a new concern, which mainly refers to a variety of neurological abnormalities, including decline in cognition, attention, memory, and psychomotor deficits caused by chronic hyperglycemia in the brain (Wang et al., 2016). Particularly, mild cognitive decline caused by diabetes mellitus has been recently highlighted as an early manifestation, a preclinical transitional state from normal cognition to dementia (Ma et al., 2015). However, to date, the precise mechanisms of cerebral dysfunction in diabetes remain unclear. Increasing evidence demonstrated that diabetes mellitus associated cerebral impairment was closely relevant with direct insult of hyperglycemia, oxidative stress, chronic inflammation, impaired insulin signaling, as well as the dementia-like pathology like amyloid polypeptide depositions and tau protein phosphorylation. These pathological processes may cause irreversible structural damages of brain, like disruption of white matter integrity and cerebral atrophy, thus contributing to cerebral dysfunction (Takeda et al., 2010; Wang et al., 2015; Yin et al., 2015; Kim et al., 2016; Tian et al., 2016; Zhang et al., 2016). Among these complicated pathogenesis, oxidative stress and inflammation were most researched and recognized for cerebral damage in diabetes (Heyer et al., 2013; Zhao et al., 2013; Moghaddam et al., 2014; Verdile et al., 2015). Hyperglycemia may activate the Toll-like receptors (TLRs), a family of pattern recognition receptors responsible for triggering the downstream inflammatory cascade (Rajamani and Jialal, 2014), thus causing neuronal lesions in the brain of chronic T2DM. In addition, high glucose and advanced glycation end products (AGEs) can initiate activation of the NADPH oxidase and trigger increased reactive oxygen species (ROS) generation, which may damage the structure and function of the brain (Zhao et al., 2013). It was recognized that nuclear factor-κB (NF-κB) signaling pathway played an important role in regulating inflammatory response and oxidative stress. Convincing data has shown that the levels of pro-inflammatory cytokines and ROS along with NF-κB activity were significantly elevated in the brain tissues of T2DM rats (Moghaddam et al., 2014). Additionally, the classical nuclear erythroid 2 related factor2 (Nrf2) signaling pathway also participated in the regulation of oxidative stress. Previous studies have reported that Nrf2 signaling pathway could protect diabetic mice against oxidative stress via mediating expressions of antioxidant proteins (Uruno et al., 2013). Under oxidative stress, Nrf2 was released from its inhibitory adaptor, Kelch-like ECH-associated protein 1 (Keap1) and translocated into the nucleus from the cytoplasm. Then, Nrf2 activated the expressions of downstream antioxidant proteins via interaction with antioxidant response element (ARE), such as heme oxygenase-1 (HO-1), superoxide dismutase (SOD), and glutathione (GSH) (Zhang et al., 2013a; FangFang et al., 2017). Thymoquinone (TQ) is a major bioactive ingredient extracted from the black cumin oil (Gholamnezhad et al., 2016), which has been widely researched for its anti-oxidative, anti-apoptotic, anticancer, and anti-inflammatory effects (Kortum et al., 2015; Thummuri et al., 2015; Guida et al., 2016; Asaduzzaman Khan et al., 2017). In addition, a large number of different researches have demonstrated that TQ could ameliorate cognitive deficits via attenuating the brain impairment induced by inflammation and oxidative stress (Bargi et al., 2017; Shao et al., 2017). MicroRNAs (miRNAs) are a kind of endogenously expressed small non-coding RNAs, which can regulate gene expression via base-pairing with the 3′-untranslated regions (3′-UTR) of target mRNA. It has recently been reported miRNAs play an essential role in various pathological processes like inflammation, immunity, oxidative stress, carcinogenesis, and so on (Kantharidis et al., 2011). Recently, increasing studies suggested that miR-146a played a vital role in inflammatory process in various disorders including diabetes (Balasubramanyam et al., 2011; Cheng et al., 2013; Li et al., 2015). In addition, miR-146a was reported to exert anti-inflammatory effect in the pathogenesis of various diabetic complications like diabetic nephropathy, retinopathy, neuropathy, cardiovascular disorders, even tending to be a potential biomarker of inflammatory status in these diseases (Yousefzadeh et al., 2015; Bhatt et al., 2016; Chen et al., 2017b; Feng et al., 2017). Moreover, it was demonstrated that the expression of miR-146a was down-regulated in hippocampus tissues of diabetic rats (Yavari et al., 2016) as well as in the serum of T2DM patients, which may serve as a biomarker of the chronic inflammatory condition (Baldeon et al., 2014). In fact, it has been uncovered that miR-146a could suppress the expressions of the NF-κB-mediated inflammatory mediators like COX-2, TNF-α, IL-6 and IL-1β by targeting the 3′-UTR of IRAK1 and TRAF6 mRNA, which are the downstream adaptors of TLRs (Taganov et al., 2006). Although considerable researches focused on neurological complications of diabetes, the basic pathogenesis remains unclear so far. Our study was designed to investigate the role of miR-146a in the processes of neuro-inflammatory and oxidative stress in the brain of chronic T2DM rats.

## Materials and Methods

### Reagents

Thymoquinone (TQ) and streptozocin (STZ) were purchased from Sigma-Aldrich Co. (St. Louis, MO, United States).

### Experimental Animals and Grouping

Male Sprague–Dawley (SD) rats weighing 160–180 g were purchased from the Animal Center of Fudan University (Shanghai, China). All animal experiments were conducted in accordance with the Guidelines for Animal Experiments of the Chinese Academy of Medical Sciences and were approved by the ethics committee for animal care of Jinshan Hospital of Fudan University. The rats were housed in a standard animal-grade room with four animals in each cage. The rats were maintained in an ambient temperature of 20 ± 2°C, the relative humidity at 60%, and a light cycle at 12 h/day. They were fed a normal laboratory diet and had free access to tap water for one week before modeling. Then, the rats were randomly divided into three groups: control (normal), chronic T2DM (cT2DM), chronic T2DM+TQ (TQ) (*n* = 12).

### Establishment of a T2DM Rat Model

Establishment of a T2DM model was previously described (Shi et al., 2013). Firstly, the rats were fed a high-fat, high-sugar diet (normal diet mixed with 10% lard and 20% sucrose) for 6 weeks. Then the diabetes model was established by a single intraperitoneal administration of STZ (35 mg/kg; Sigma) in 0.1 M citrate buffer (pH 4.2) after overnight fasting. Diabetes was validated by measuring blood glucose levels (>16.7 mmol/L) 72 h after STZ injection. Once the diabetes was induced, the diabetic animals were divided into chronic T2DM group and chronic T2DM +TQ group. The chronic T2DM group were continuously fed with a high-fat, high-sugar diet for another 6 weeks. But the TQ group animals were intraperitoneally injected with 5 mg/kg TQ (274666, Sigma; dissolved in 10% anhydrous ethanol), once every 2 days, except with a high-fat, high-sugar diet for 6 weeks. The normal control group rats were given the normal laboratory diet all the time in the experiment and intraperitoneally injected with equivalent volume of normal saline.

### Quantitative Real-Time PCR Analysis

Total RNA was isolated from the brain tissues by TRIZOL Reagent (Takara) according to the manufacturer’s protocol. Then, cDNA of miR-146a was synthesized by Mir-X miRNA First-Strand Synthesis Kit (Takara, Japan) while the cDNA of mRNA for target genes including p22phox, IRAK1, TRAF6, and NF-kB p65, was synthesized by PrimeScript ^TM^ RT Master Mix (Takara, Japan). Subsequently, quantitative real-time PCR (qRT-PCR) of miR-146a was performed with the Mir-X miRNA qRT-PCR SYBR Kit (Takara, Japan) in Applied Biosystem 7300 (Applied Biosystems, Foster city, CA, United States). The expression level of miR-146a was determined using 2^-ΔΔCt^ and normalized using U6 snRNA level as an internal quantitative control. For real-time measurement of mRNAs, a SYBR Premix Ex Taq (Tli RNaseH Plus; TaKaRa) was used for detecting expression level of β-actin and respective target genes. The expression level of mRNA was determined using 2^-ΔΔCt^ and normalized to β-actin.

### Western Blot Assay

Total protein was extracted from brain tissues with a SDS lysis buffer (Beyotime, Shanghai, China), supplemented with 1% phenylmethylsulfonyl fluoride (Beyotime, Shanghai, China). Equal amount of proteins was analyzed by 10% SDS–PAGE and transferred to PVDF membranes. After being blocked in 5% non-fat milk at room temperature for 1 h, the membranes were incubated with primary antibodies at 4°C overnight, including rabbit anti-COX-2 antibody (Cell Signaling, United States), rabbit anti-TNF-α antibody (Millipore, United States), rabbit anti-IL-1β antibody (Abcam, United States), rabbit anti-p-NF-κB (Cell Signaling, United States), rabbit anti-TRAF6 antibody (Proteintech group, United States), mouse anti-IRAK1 antibody and rabbit anti-Nrf2 antibody (Santa Cruz Biotechnology, United States), rabbit anti-HO-1 antibody (Abcam, United States), and rabbit anti-β-actin antibody (Cell Signaling, United States). The appropriate peroxidase-conjugated antibodies, anti-mouse, or anti-rabbit were incubated with the membranes at room temperature for 2 h. Signals were detected using ECL-Plus (Merck Millipore, Darmstadt, Germany) and quantified using a Bio-Rad 2000 gel imaging system with QUANTITY ONE software (Bio-Rad Laboratories, Hercules, CA, United States).

### Assay of Super Oxide Dismutase (SOD)

The level of SOD in the cerebral cortex and hippocampal tissues was quantified using a SOD kit (Fujian Fuyuan Biological Technology Co., Ltd., Fujian, China). The 40-mg sample of brain tissues was weighed and added to 360 μl of physiological saline. The mixture was homogenized using a vortex mixer and centrifuged (1,000 × *g*, 4 °C, 10 min) to obtain the supernatant, which was used to detect the level of SOD according to the manufacturer’s instruction.

### Malondialdehyde (MDA) Assessment

Malondialdehyde (MDA) was assayed using MDA kit (Nanjing Jiancheng Bioengineering Institute, Nanjing, China), which is based on MDA reaction with thiobarbituric acid (TBA) and producing a pink complex with a peak absorbance at 535 nm. The protocol of MDA assessment was previously described (Bargi et al., 2017). The 40-mg sample of brain tissues was weighed and added to 360 μl of physiological saline. Then the mixture was homogenized using a vortex mixer and centrifuged to obtain the supernatant, which was used to measure the MDA level according to the manufacturer’s instruction.

### Statistical Analysis

Statistical analyses were performed using SPSS 17.0 (SSPS, Inc., Chicago, IL, United States) as well as a one-way ANOVA or Pearson correlation analysis. Values are expressed as the mean ± SD, and statistical significance was defined as *p* < 0.05 for all tests.

## Results

### Aberrant Morphological Changes in the Brain of Chronic T2DM Rats

**FIGURE 1:**
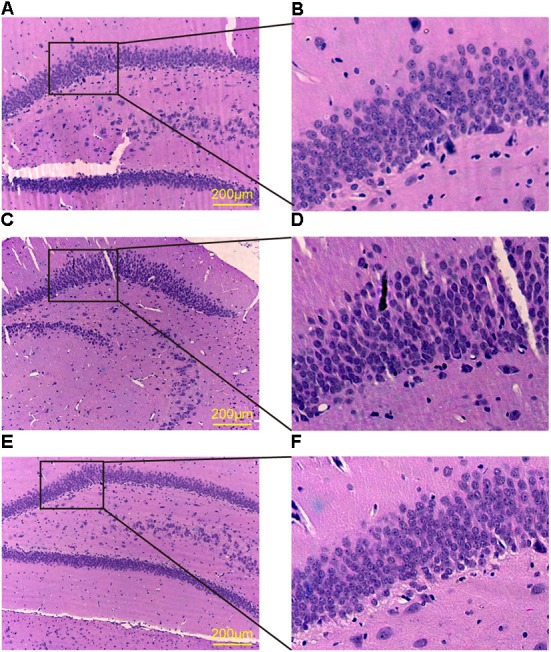
HE staining of the dentate gyrus in the hippocampus. Normal group **(A,B)**; cT2DM group **(C,D)**; TQ group **(E,F)**.

It has been widely accepted that the hippocampal synapse impairment contributes to the cognitive deficits (Zhang et al., 2013b). In the present study, H&E staining was performed to observe the morphological changes of the rat hippocampus. The neurons in the hippocampus of cT2DM group showed loose and swollen, which were irregular and disorganized compared with the normal group. Meanwhile, karyopyknosis was obviously observed in the neurons of dentate gyrus of the cT2DM group (**Figures 1A–D**). The morphological dysfunction could be ameliorated in the TQ group compared to the cT2DM group (**Figures 1E,F**).

### Increased Inflammatory Mediators in the Brain of Chronic T2DM Rats

**FIGURE 2:**
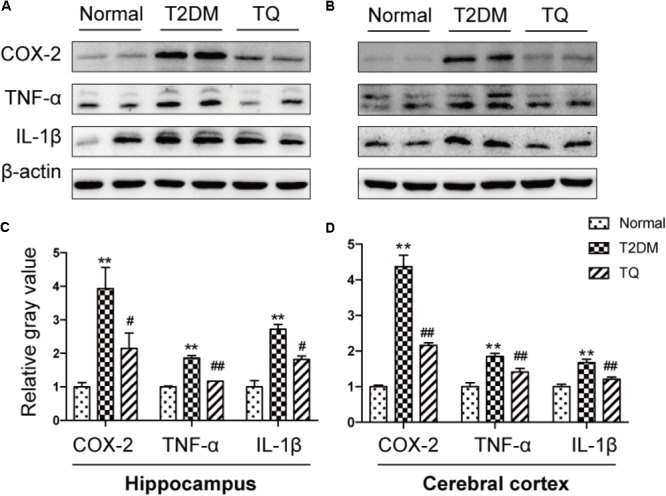
Representative protein bands of COX-2, TNF-α, and IL-1β in the hippocampus **(A)** and cerebral cortex **(B)**; the relative ratio COX-2, TNF-α, and IL-1β according to band density in the hippocampus **(C)** and cerebral cortex **(D)**. ^∗^*p* < 0.05 vs. normal; ^∗∗^*p* < 0.01 vs. normal; ^#^*p* < 0.05 vs. cT2DM; ^##^*p* < 0.01 vs. cT2DM.

Growing evidence indicated that inflammation and its accompanied stress responses contributed to cognitive decline in cT2DM rats (Chen et al., 2017a). To determine inflammatory status in the brain of cT2DM rats, we detected the expressions of classical inflammatory mediators in the hippocampus and cerebral cortex. The levels of COX-2, TNF-α and IL-1β were significantly augmented in the hippocampus (**Figures 2A,C**) and cerebral cortex (**Figures 2B,D**) of cT2DM group compared with the normal group (^∗^*p* < 0.05 vs. normal; ^∗∗^*p* < 0.01 vs. normal). In contrast, TQ could downregulate the expressions of these inflammatory mediators (^#^*p* < 0.05 vs. cT2DM; ^##^*p* < 0.01 vs. cT2DM).

### Increased Oxidative Stress Status in the Brain of Chronic T2DM Rats

**FIGURE 3:**
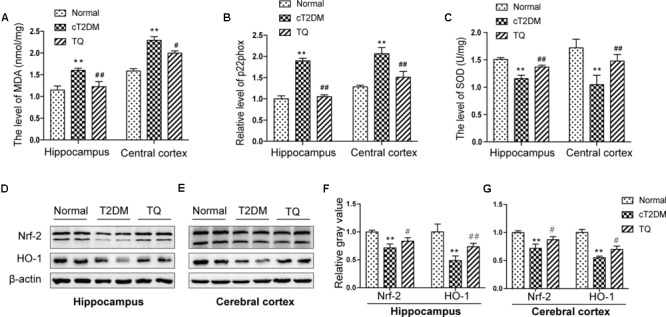
The expressions of MDA **(A)**, p22phox **(B)**, and SOD **(C)** in the hippocampus and cerebral cortex. Representative protein bands of Nrf2 and HO-1 in hippocampus **(D)** and cerebral cortex **(E)**; the relative ratio of Nrf2 and HO-1according to band density in hippocampus **(F)** and cerebral cortex **(G)**. ^∗^*p* < 0.05 vs. normal; ^∗∗^*p* < 0.01 vs. normal; ^#^*p* < 0.05 vs. cT2DM; ^##^*p* < 0.01 vs. cT2DM.

Recently, a substantial literature has implied that oxidative stress, which is induced for the imbalance between ROS production and the anti-oxidative system, could exaggerate the pathological lesions in the brain of cT2DM rats (Uruno et al., 2013). In the study, to evaluate the level of oxidative stress, we assayed the expressions of oxidant indicators including p22phox and MDA in the hippocampus and cerebral cortex of cT2DM rats. p22phox is a subunit of NADPH oxidase, which is served as a major source of ROS. As showed in **Figures 3A,B**, the levels of MDA and p22phox increased in the brain of cT2DM group (^∗^*p* < 0.05 vs. normal; ^∗∗^*p* < 0.01 vs. Normal) while decreased in TQ group (^#^*p* < 0.05 vs. cT2DM; ^##^*p* < 0.01 vs. cT2DM). In addition, the levels of Nrf2, HO-1, and SOD were measured to determine the antioxidant condition in the brain of cT2DM models. Our results showed that the levels of Nrf2, HO-1, and SOD significantly decreased in the brain of cT2DM group (^∗^*p* < 0.05 vs. normal; ^∗∗^*p* < 0.01 vs. normal) while increased in the TQ group (^#^*p* < 0.05 vs. cT2DM; ^##^*p* < 0.01 vs. cT2DM) (**Figures 3C–G**).

### Analysis of the Correlations Between miR-146a and Inflammation Status

**FIGURE 4:**
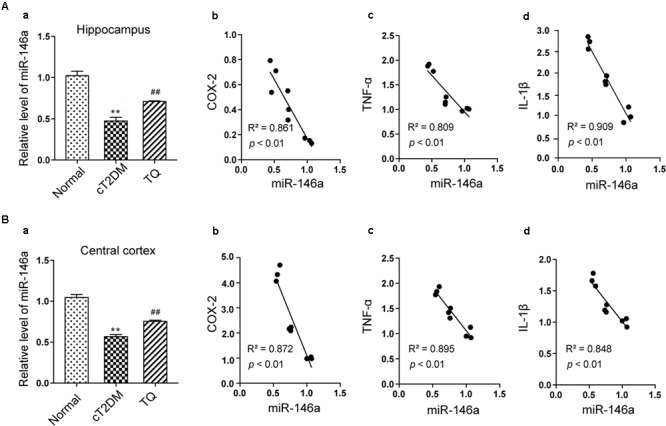
Analysis of the correlations between miR-146a and inflammatory mediators. The level of miR-146a in the hippocampus **(Aa)** and cerebral cortex tissues **(Ba)** by qRT-PCR. In the hippocampus, the correlations between miR-146a level and the expressions of COX-2 **(Ab)**, (*R*^2^ = 0.861, *p* < 0.01), TNF-α **(Ac)**, (*R*^2^ = 0.809, *p* < 0.01), and IL-1β **(Ad)**, (*R*^2^ = 0.909, *p* < 0.01). In the cerebral cortex, the correlations between miR-146a level and the expressions of COX-2 **(Bb)**, (*R*^2^ = 0.872, *p* < 0.01), TNF-α **(Bc)**, (*R*^2^ = 0.895, *p* < 0.01), and IL-1β **(Bd)**, (*R*^2^ = 0.848, *p* < 0.01). ^∗∗^*p* < 0.01 vs. Normal; ^##^*p* < 0.01 vs. cT2DM.

Convincing data has shown that miR-146a plays a vital role in inflammatory process in various disorders including diabetes mellitus (Balasubramanyam et al., 2011). In this study, the expression of miRNA-146a significantly decreased in cT2DM group (^∗∗^*p* < 0.01 vs. normal) while increased in TQ group (^##^*p* < 0.01 vs. cT2DM) both in the hippocampus (**Figures 4Aa**) and cerebral cortex tissues (**Figures 4Ba**). Then, Pearson linear measurement was conducted to investigate the relationship between miR-146a and inflammatory mediators. There existed negative correlations between miR-146a and inflammatory mediators including COX-2, TNF-α, and IL-1β in the hippocampus of cT2DM group (**Figures 4Ab–d** *R*^2^ = 0.861, *p* < 0.01; *R*^2^ = 0.809, *p* < 0.01; *R*^2^ = 0.909, *p* < 0.01, respectively) and in the cerebral cortex (**Figures 4Bb–d** *R*^2^ = 0.872, *p* < 0.01; *R*^2^ = 0.895, *p* < 0.01; *R*^2^ = 0.848, *p* < 0.01; respectively). Pearson correlation analysis demonstrated that the change in miR-146a was negatively correlated with the levels of inflammatory mediators.

### Analysis of the Correlation Between miR-146a and Oxidative Stress Status

**FIGURE 5:**
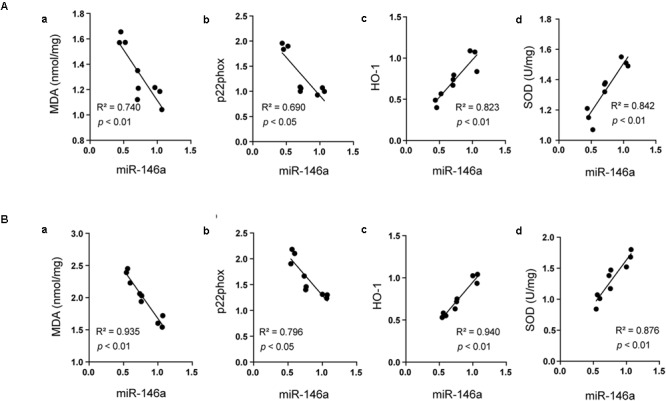
Analysis of the correlation between miR-146a and oxidative status. The correlations between miR-146a level and the expressions of MDA **(Aa)** (*R*^2^ = 0.740, *p* < 0.01), p22phox **(Ab)**, (*R*^2^ = 0.690, *p* < 0.05), HO-1 **(Ac)**, (*R*^2^ = 0.823, *p* < 0.01), and SOD **(Ad)**, (*R*^2^ = 0.842, *p* < 0.01) in the hippocampus. The correlations between miR-146a level and the expressions of MDA **(Ba)**, (*R*^2^ = 0.935, *p* < 0.01), p22phox **(Bb)**, (*R*^2^ = 0.796, *p* < 0.05), HO-1 **(Bc)**, (*R*^2^ = 0.940, *p* < 0.01), and SOD **(Bd)**, (*R*^2^ = 0.876, *p* < 0.01) in the cerebral cortex.

Recent studies highlighted that miR-146a was closely involved in oxidative stress induced by hyperglycemia (Wan and Li, 2018). In the current study, we assayed the correlations between the levels of miR-146a and oxidant indicators as well as antioxidant proteins using Pearson linear measurement. We observed that change in miR-146a was negatively correlated with the levels of oxidant indicators including MDA and p22phox in the hippocampus (**Figures 5Aa,b** *R*^2^ = 0.740, *p* < 0.01; *R*^2^ = 0.690, *p* < 0.05) and in the cerebral cortex (**Figures 5Ba,b** *R*^2^ = 0.935, *p* < 0.01; *R*^2^ = 0.796, *p* < 0.05). While the level of miR-146a was positively correlated with the antioxidant proteins including HO-1 and SOD in the hippocampus (**Figures 5Ac,d** *R*^2^ = 0.823, *p* < 0.01; *R*^2^ = 0. 842, *p* < 0.01) and in cerebral cortex (**Figures 5Bc,d** *R*^2^ = 0.94, *p* < 0.01; *R*^2^ = 0.876, *p* < 0.01).

### MiR-146a Might Regulate Inflammation and Oxidative Stress Status via NF-κB Signaling Pathway

**FIGURE 6:**
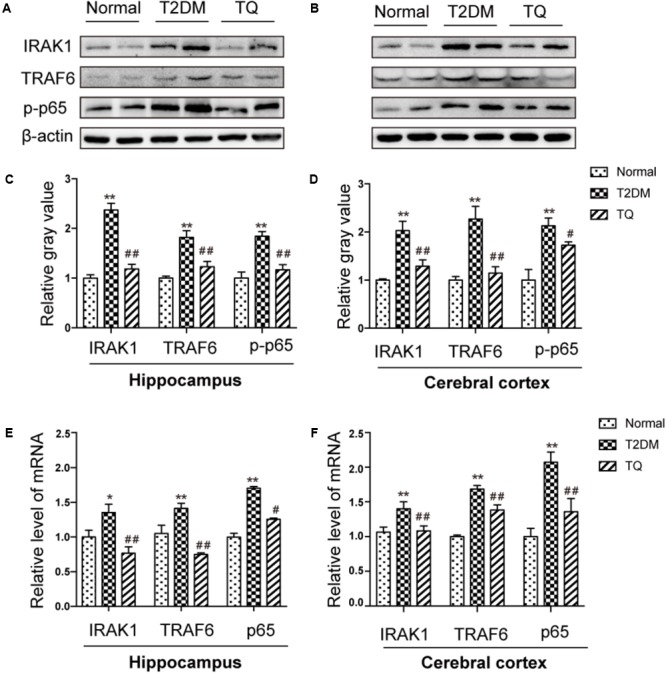
Representative protein bands of IRAK1, TRAF6, and NF-κB p-p65 in hippocampus **(A)** and cerebral cortex **(C)**. The relative ratio of IRAK1, TRAF6, and NF-κB p-p65 according to band density in hippocampus **(B)** and cerebral cortex **(D)**. The mRNA expression levels of IRAK1, TRAF6 and NF-kB p65 in the hippocampus **(E)** and in the cerebral cortex **(F)**. ^∗^*p* < 0.05 vs. normal; ^∗∗^*p* < 0.01 vs. normal; ^#^*p* < 0.05 vs. cT2DM; ^##^*p* < 0.01 vs. cT2DM.

It has been reported that miR-146a could inhibit NF-κB-mediated signaling pathway by suppressing its target genes, including IRAK1 and TRAF6, which was closely involved in inflammatory and oxidative processes (Liu et al., 2017). Then, our results showed the expressions of IRAK-1, TRAF6, and phosphorylated NF-κB p65 markedly increased in cT2DM group (^∗∗^*p* < 0.01 vs. normal) while decreased in TQ group (^##^*p* < 0.01 vs. cT2DM) by western blot in the hippocampus (**Figures 6A,C**) and in the cerebral cortex (**Figures 6B,D**). In addition, the results of EMSA showed increased activation of NF-κB in the cerebral cortex of cT2DM rats compared with the normal rats (**Supplementary Figure S1**). We also examined the mRNA levels of IRAK-1, TRAF6, and NF-κB p65 using qRT- PCR and the results showed the similar changes with the protein expressions (**Figures 6E,F**), which were negatively correlated with the miR-146a level. Our results indicated that miR-146a might regulate the inflammation and oxidative stress status in the brain of cT2DM models via NF-κB signaling pathway.

## Discussion

**FIGURE 7:**
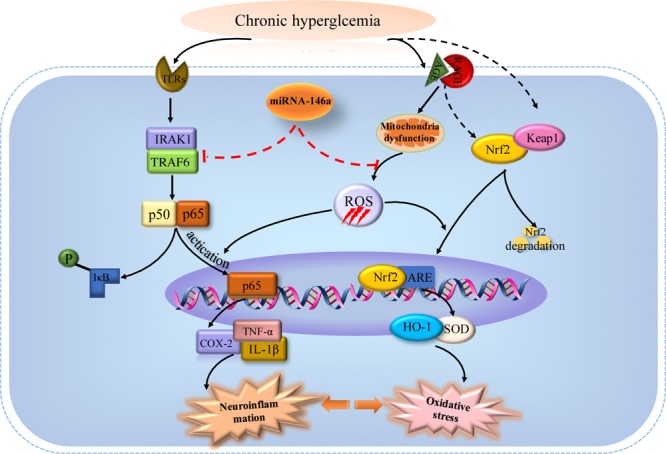
The potential mechanisms of miRNA-146a in regulating inflammation and oxidative stress in the brain of chronic T2DM models (activation steps are represented by solid lines and inhibitory effects are represented by dashed lines).

Diabetes-related brain impairment, mainly manifested as cognitive and behavior dysfunction, has recently become a hot spot of attention in patients with long-term T2DM (Sima, 2010). In fact, accumulating literatures highlighted that diabetic patients suffered from higher risk of developing Alzheimer’s disease (AD) and other type of dementia (Moreira, 2012). Previous investigations indicated that chronic inflammation and oxidative stress are the two key factors connecting T2DM to AD via impairing cerebral insulin signaling and disturbing amyloid-β (Aβ) protein metabolism (Verdile et al., 2015). Hyperglycemia may activate several signaling adaptor proteins, like protein kinase C (PKC) and TLRs, thus triggering the downstream NF-κB-mediated inflammatory cascade (Rajamani and Jialal, 2014). In addition, high glucose and AGEs could disturb mitochondrial metabolism and aggravate ROS production via triggering the activation of NADPH oxidase. A large amount of ROS could lead to a further activation of the NF-κB signaling pathway, ultimately exacerbating oxidative stress in diabetic patients (Morcos et al., 2008; Xiang et al., 2012; Muriach et al., 2014). Furthermore, the processes of oxidative stress and inflammation are interdependent, which can be induced and further enhanced with each other, thus exacerbating the brain insult in T2DM (Biswas, 2016). In detail, inflammatory cells could respond to oxidative stress by releasing various NF-κB-mediated pro-inflammatory mediators, which in turn aggravated the status of inflammation and oxidative stress, thereby establishing a vicious cycle. A common hypothesis for the cause of the cerebral insult in diabetes associated oxidative stress with inflammation via NF-κB, a key regulator of inflammation as well as a potent sensor of oxidative stress. NF-κB may play an essential role at the crossroad between oxidative stress and inflammation (Muriach et al., 2014). Consistent with previous studies, we also found that the levels of inflammatory mediators like TNF-α, COX-2, IL-1β along with phosphorylated NF-κB p65 dramatically increased in the brain tissues of T2DM rats (**Figure 2**). Meanwhile, the redox homeostasis was disturbed because of increased expressions of pro-oxidant molecules (MDA and p22phox) and decreased expressions of antioxidant proteins like HO-1 and SOD in the brain of cT2DM rats (**Figure 3**). Substantial literature has confirmed that TQ has anti-oxidative and anti-inflammatory functions (Woo et al., 2012; Al Wafai, 2013). In our study, we found that TQ could inhibit the expressions of inflammatory mediators and alleviate the oxidative stress status as well as the activation of NF-κB p65. Therefore, our results indicated that TQ may attenuate the inflammatory and oxidative stress processes via inhibiting NF-κB signaling pathway. Furthermore, the emerging study has demonstrated that TQ inhibited NF-κB-mediated neuro-inflammation dependent on activating Nrf2-ARE signaling pathway, whereas silencing Nrf2 expression resulted in the loss of anti-inflammatory effects (Velagapudi et al., 2017). In contrast, new findings have found that NF-κB could repress the Nrf2-ARE pathway through interaction with Keap1 (Yu et al., 2011; Sandberg et al., 2014). Therefore, the relationship between anti-inflammatory effect and anti-oxidative effect of TQ may be interdependent. Inflammation and oxidative stress are tightly linked and interdependent pathophysiological processes. Therefore, antioxidant therapy alone was not sufficient to prevent diseases induced by oxidative stress, which explained why Nrf2-mediated neuroprotection failed to prevent cognitive decline in diabetes (McNeilly et al., 2016). Interestingly, TQ relieved the inflammation and oxidative stress status accompanied with increased expression of miR-146a. Recently, miR-146a was recognized as a potent regulator in inflammatory reaction in various diseases associated with inflammation and oxidative stress (Su et al., 2016; Wan and Li, 2018). It has been uncovered that the level of miR-146a was reversely associated with chronic inflammatory and oxidative processes via a negative feedback regulation. The expression of miR-146a can be induced by pro-inflammatory factors and ROS via activating the transcription factor NF-κB, which located at the upstream of the promoter of miR-146a. In turn, miR-146a could inhibit the activation of NF-κB mediated inflammation via suppressing its target genes expression like IRAK1 and TRAF6 (Li et al., 2015; Lo et al., 2017). Knock-out of miR-146a in the mice under diabetic condition resulted in increased pro-inflammatory phenotype and macrophage infiltration (Bhatt et al., 2016). Furthermore, it was verified that miR-146a could alleviate hyperglycemia induced endothelial inflammation by inhibiting NAPDH oxidase 4 expression (Wang et al., 2014a). Consistent with previous studies, our study also found the level of miR-146a decreased along with increased level of inflammation in the brain of chronic T2DM rats, while in the TQ group, miR-146a level increased along with reduced inflammation and oxidative stress status. It implied that the level of miR-146a was elevated because of the decreased status of inflammation and oxidative stress, which tended to be a credible comprehensive indicator of inflammation and oxidative stress. However, there is doubt that whether TQ exerted anti-inflammatory and anti-oxidative effects via increasing the expression of miR-146a, which could suppress the NF-κB signaling pathway. Further investigation is needed to test the hypothesis. In addition, miR-146a expression was found obviously decreased in the serum of subjects with T2DM as well as other tissues affected by chronic hyperglycemia like heart, retina, dorsal root ganglion neurons, and hippocampus, which was negatively correlated to the inflammatory state (Wang et al., 2014b). Paradoxically, the level of miR-146a was found increased in the sciatic nerve and kidney tissues in chronic T2DM rats. The difference may be explained by the fact that the expression of miR-146a is dependent on the tissue type, duration of diabetes, and gene polymorphism apart from severity of inflammation (Kaidonis et al., 2016). Overall, our results indicated that increased status of inflammation and oxidative stress contributed to brain impairment in cT2DM rats, which may be negatively regulated by miR-146a. In addition, the level of miR-146a in the brain may indirectly serve as a negative biomarker of the severity of chronic inflammation and oxidative stress in the brain of cT2DM rats (**Figure 7**). However, there were some disadvantages in our study because we did not directly intervene miR-146a expression to observe its impact on inflammation and oxidative stress. Therefore, further research is needed to detect the role of miR-146a in regulating inflammation and oxidative stress in brain impairment of T2DM rats.

## Author Contributions

YX, AC, and YF performed most of the experiments and wrote the manuscript. YS, QL, and XD participated in statistical analysis. LC and MW helped conduct the establishment of T2DM models. XS and YC contributed to the design and performance of experiments. All authors have read and approved the final manuscript.

## Conflict of Interest Statement

The authors declare that the research was conducted in the absence of any commercial or financial relationships that could be construed as a potential conflict of interest.

## Supplementary Material

FIGURE S1 Increased NF-κB activation in the cerebral cortex of cT2DM rats. The activation level of p65 increased in the cerebral cortex of cT2DM rats compared with the normal group but significantly decreased in the TQ group by EMSA.
