# Supplementary figures and images for: MicroRNA-146a: A Comprehensive Indicator of Inflammation and Oxidative Stress Status Induced in the Brain of Chronic T2DM Rats
Source: Front Pharmacol. 2018 May 14;9:478. doi: 10.3389/fphar.2018.00478 (PMC5960742; doi:10.3389/fphar.2018.00478)

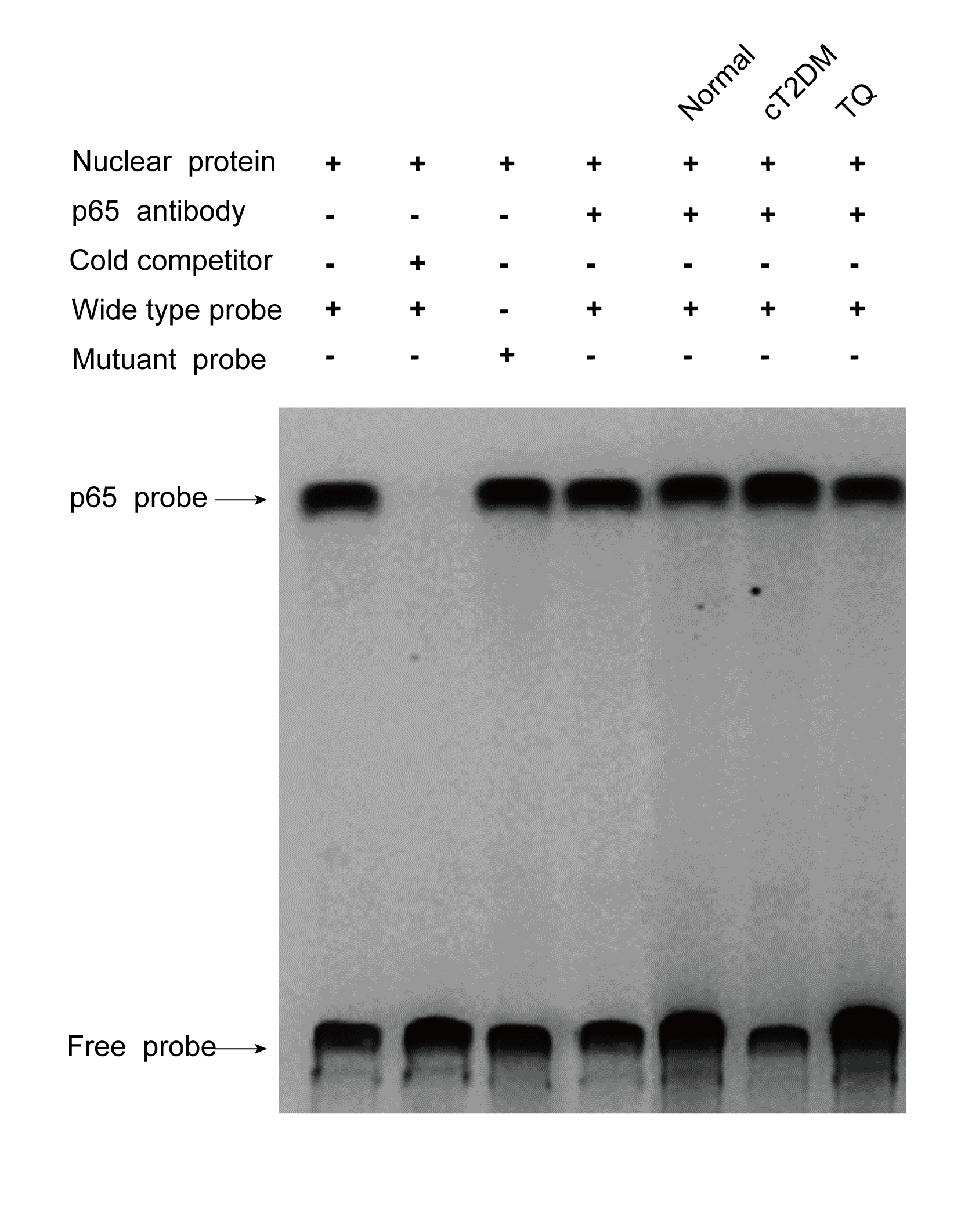

Supplement: FIGURE S1 — Increased NF-κB activation in the cerebral cortex of cT2DM rats. The activation level of p65 increased in the cerebral cortex of cT2DM rats compared with the normal group but significantly decreased in the TQ group by EMSA. [file Image_1.TIF]
